# Association between lifestyle factors and metabolic syndrome in general populations with depressive symptoms in cross-setional based cohort study of Ansung-Ansan

**DOI:** 10.1371/journal.pone.0262526

**Published:** 2022-03-15

**Authors:** Jinhee Lee, Tae Hwa Go, Seongho Min, Sang Baek Koh, Jung Ran Choi

**Affiliations:** 1 Department of Psychiatry, Yonsei University Wonju College of Medicine, Wonju, Republic of Korea; 2 Center of Biomedical Data Science, Yonsei University Wonju College of Medicine Wonju, Republic of Korea; 3 Institute of Genomic Cohort, Yonsei University Wonju College of Medicine, Wonju, Republic of Korea; 4 Department of Preventive Medicine, Yonsei University Wonju College of Medicine, Wonju, Republic of Korea; Health Services Academy, PAKISTAN

## Abstract

**Background:**

Metabolic syndrome (MetS) is caused by both genetic and environmental factors, such as daily calorie intake, smoking, and alcohol consumption. Lifestyle factors, such as alcohol consumption, are considered to be related to the prevalence of MetS and plays an essential role in the pathogenesis and prognosis of depression.

**Methods:**

We investigated the bidirectional association between lifestyle factors and MetS among Korean adults with depressive symptoms in third wave of a community-based cohort study. A total of 1,578 individuals, aged 39–72 years, who had MetS at baseline were recruited. Participants were divided into two groups according to depressive symptoms. Logistic regression models were used to estimate the risk of MetS.

**Results:**

The percentage of heavy drinkers was lower in men with depressive symptoms compared to those who did not (7.0% vs. 7.1%), while the percentage of current smokers were higher in participants who had depressive symptoms (40.2% vs. 30.0%). After adjusting for age, education, monthly income, body mass index (BMI), sleep duration, and volume of drinking and smoking status, logistic regression analysis demonstrated that male heavy drinkers with depressive symptoms were 2.75 times more likely to have MetS than those without depressive symptom. Conversely, depressive women with a high BMI were 3.70 times more likely to have MetS than in those with lower BMI. Limitations The cross-sectional nature of the study, and the study population ethnicity and ages were limitations.

**Conclusions:**

Lifestyle factors, such as alcohol consumption, may be associated with the risk of MetS in adults with depressive symptoms.

## Introduction

Depression is one of the most common mental disorders in most countries and regions, and the number of depressed adults has increased substantially in recent years. Frequencies of depression and subsyndromal depressive indications are reported as 1–4% and 10–15%, respectively [1]. Depression is associated with increased risk of morbidity, decreased physical activity, and cognitive functioning, all of which are in turn associated with an increased risk of metabolic syndrome (MetS) [2]. A meta-analysis showed that individuals with depression had 1.5 times higher odds of having MetS [3]. There are several hypotheses that depression and MetS may share common mechanisms underlying their association. A number of prior studies have indicated that MetS and depression might cause common changes in stress-related systems such as the hypothalamus–pituitary–adrenal (HPA) axis, the autonomic nervous system, and the immune system, and also affect platelet and endothelial function [4]. Metabolic syndrome is also known to be associated with the unhealthy lifestyle habits of depressed patients. Recently, Pan A et al. found that depression and MetS were significantly correlated in cross-sectional studies, and a bidirectional association was observed in prospective cohort studies.

Metabolic syndrome is a complex of interconnected risk factors such as abdominal obesity, elevated blood pressure, glucose intolerance, hyperglycemia, and low high-density lipoprotein (HDL) cholesterol levels [5, 6]. The syndrome is an essential health problem worldwide, and associated with the risk of atherosclerotic cardiovascular disease, type 2 diabetes mellitus and cancer [6, 7]. Previous studies have confirmed that lifestyle interventions, including diet control, weight loss, exercise intervention, and their combinations, improve components of MetS in the general population [8].

Patients with depression are at high risk of developing MetS because of the negative effects associated with components of MetS, including unhealthy diet habits, high consumption of alcohol, and poor physical activity due to low mood [4]. Lifestyle modifications, such as exercise, dietary changes, smoking cessation, and reducing alcohol intake may play an important role in reducing MetS morbidity and also in improving symptoms of depression.

Thus, this study aims to identify which lifestyle factors contribute to the bidirectional association between MetS and depression. The study examined the prevalence of MetS and assessed the association between lifestyle factors and the risk of MetS in patients with symptoms of depression.

## Methods

### Study population

This study was performed using data from the Korean Genome and Epidemiology Study on Urban and Rural Community in the Korean General Population (KoGES, Ansung, and Ansan), which was conducted to evaluate incidence and risk factors for chronic diseases, such as diabetes mellitus and hypertension [9]. All adults dwelling in rural areas of Ansung and urban areas of Ansan in South Korea, where demographic shifts are infrequent and the population can be followed long-term, were recruited for this study.

**Fig 1:**
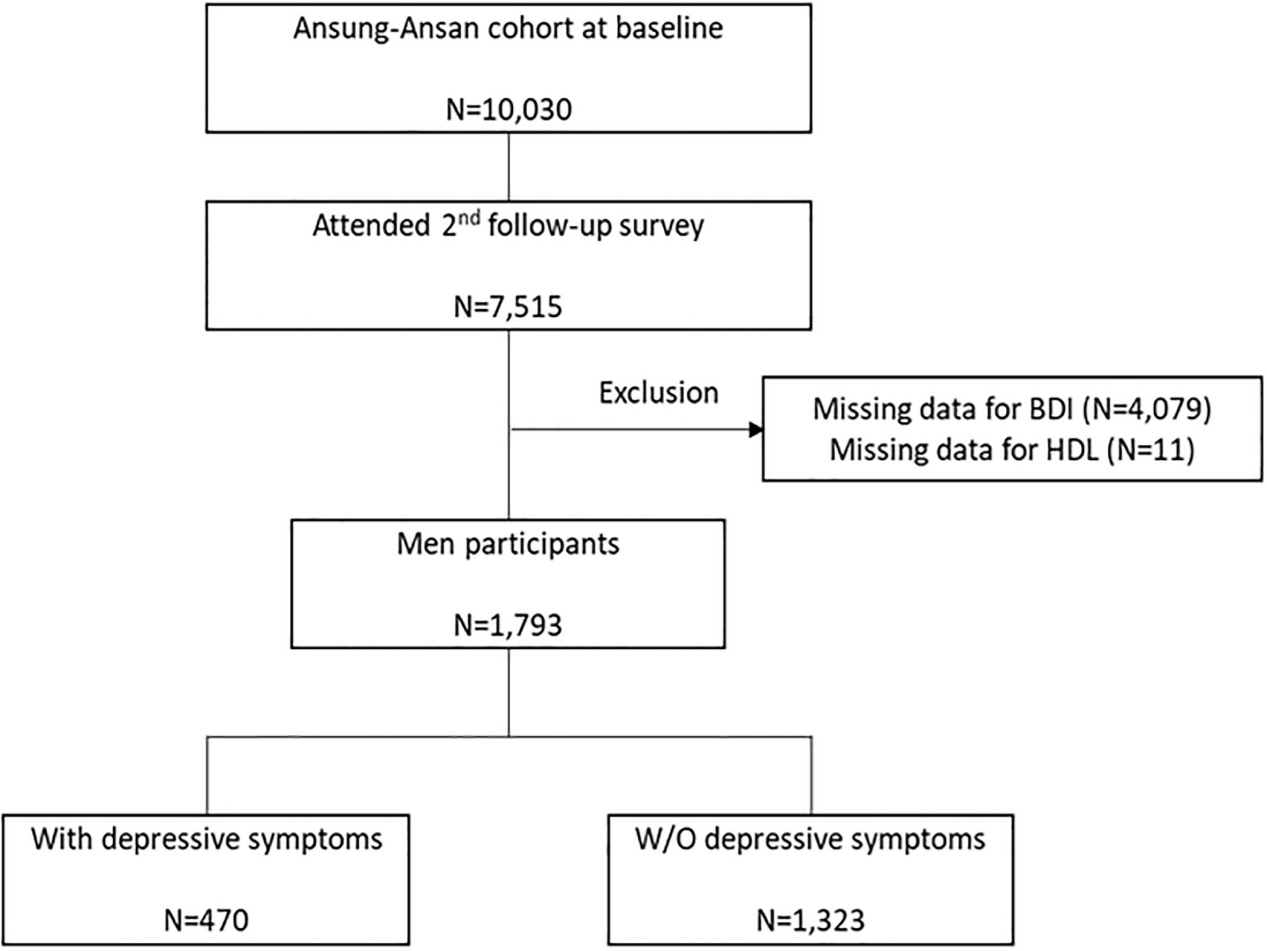
Flow chart showing the study population selection.

The baseline survey, carried out from 2001 to 2002, included 10,030 adults aged 40–69 years, and a total of seven traces were investigated. The Beck Depression Inventory (BDI) was only investigated in the second follow-up survey. We included 7,515 individuals who participated in the second follow-up survey. Among them, we excluded 4,079 participants with missing BDI data and 11 participants with missing HDL cholesterol data. After exclusion, 1,793 men and 1,632 women were eligible for this study (Fig 1). The study protocol was approved by the Institutional Review Board of Ajou University Medical Center and the Korea University Ansan Hospital. All participants provided written informed consent.

### Definition of variables

Age was treated as a continuous variable. Education level was divided into middle school or less or high school or more, and income level was divided into mean monthly household income < 1,500,000 won or ≥ 1,500,000 won. Body mass index was categorized as < 25 kg/m^2^ and ≥ 25 kg/m^2^ based on WHO Asia-Pacific criteria. Exercise and sleep duration were divided into < 150 min/week or ≥ 150 min/week and < 7 hour/day or ≥ 7 hour/day, respectively. Drinking status was divided into never, past, and current, and volume of drinking was divided into groups of <200 g/week, 200–400 g/week, and ≥400 g/week. Smoking status was categorized as never smoked, ex-smoker, and current smoker. Percentages of total caloric intake from carbohydrates, protein and fat were categorized as low (0–25%), middle (25–75%), and high (75–100%), considering the quartiles.

### Definition of metabolic syndrome

MetS was defined as the presence of at least 3 of the following criteria: 1) Abdominal obesity, defined as a waist circumference≥90 cm for men or≥85 cm for women (following the Korean-specific cutoffs for abdominal obesity defined by the Korean Society of Obesity) [10]; 2) Hypertriglyceridemia, defined as a serum triglyceride concentration of ≥150 mg/dl (1.69 mmol/l); 3) Low HDL cholesterol, defined as a serum HDL cholesterol concentration < 40 mg/dl (1.04 mmol/l) for men or < 50 mg/dl (1.29 mmol/l) for women; 4) High blood pressure, defined as a SBP of ≥130 mmHg or a diastolic blood pressure≥85 mmHg, or treatment with antihypertensive agents; and 5) High fasting glucose, defined as a fasting serum glucose ≥100 mg/dl or previously diagnosed T2DM [11].

### Statistical analysis

The Student’s t-test and Pearson’s Chi-squared test were used to confirm the difference in anthropometric characteristics according to depressive symptoms. Continuous variables are presented as mean ± standard deviation, and categorical variables are expressed as numbers and percentages. Multivariate logistic regression was used to evaluate the independent association between alcohol consumption and MetS in participants with and without depressive symptoms. Adjusted variables were age, education, monthly income, BMI, exercise group, sleep duration, drinking status or volume of drinking, alcohol consumption, smoking status, and total caloric intake from carbohydrates, protein, and fat. Results were expressed as odds ratios with 95% confidence intervals (CI). All analyses were performed using SAS version 9.4 (SAS Institute, Cary, NC, USA). *P*-values <0.05 were considered statistically significant.

## Results

Body mass index and WC were significantly lower in men with depressive symptoms compared to those without; conversely, triglycerides and diastolic blood pressure (DBP) were higher in women who had depressive symptoms than those who did not (Table 1). However, these differences were not statistically significant (Table 1).

**Table 1:** Metabolic syndrome components of subjects with and without depressive symptoms.

Variable	Men			Women		
	With depressive symptoms	Without depressive symptoms	p-value	With depressive symptoms	Without depressive symptoms	p-value
Total, n (%)	470	1323		616	1016	
MetS	126 (26.8)	314 (25.8)	0.6609	151 (24.5)	214 (21.1)	0.1049
WC	83.3±7.4	84.2±7.0	0.0169	79.0±8.0	78.7±8.0	0.4329
TG	159.9±114.0	152.7±110.2	0.2275	124.1±65.7	122.4±79.8	0.6375
HDL	42.7±9.8	43.0±10.1	0.6101	47.2±10.1	47.0±10.4	0.7136
SBP	116.4±15.0	117.2±14.8	0.3449	113.0±157	111.5±15.4	0.0534
DBP	79.0±10.5	79.3±10.0	0.5988	74.0±9.9	73.9±10.1	0.9300
FBG	94.5±18.2	93.9±14.1	0.5772	88.0±9.4	88.3±14.5	0.5847
BMI	24.1±2.8	24.6±2.6	0.0007	24.4±2.9	24.6±3.1	0.4328

MetS: metabolic syndrome, WC: waist circumference, TG: triglyceride, HDL: high-density lipoprotein cholesterol, SBP: systolic blood pressure, DBP: diastolic blood pressure, FBG: fasting blood glucose, BMI: body mass index.

Among the study population, the percentage of heavy drinkers was lower in men with depressive symptoms compared to those who did not (7.0% vs. 7.1%), while the percentage of current smokers was higher in participants who had depressive symptoms (40.2% vs. 30.0%). Meanwhile, the percentage of those with sleep duration ≥7 hr/day was lower in women with depressive symptoms when compared to those without (Table 2).

**Table 2:** Sociodemographic characteristics of subjects with and without depressive symptoms.

	Men			Women		
Variable	With depressive symptoms	Without depressive symptoms	p-value	With depressive symptoms	Without depressive symptoms	p-value
Total, N (%)	470	1323		616	1016	
Age	52.8±7.8	52.1±7.1	0.1112	54.2±8.0	52.1±7.4	< .0001
Education, N (%)			0.1560			0.0001
Middle school or less	46 (9.8)	102 (7.7)		177 (28.7)	208 (20.5)	
High school or more	423 (90.2)	1221 (92.3)		439 (71.3)	807 (79.5)	
Monthly income, N (%)			< .0001			< .0001
<1,500,000	189 (14.3)	1077 (22.8)		217 (35.5)	247 (24.4)	
≥1,500,000	1129 (85.7)	363 (77.2)		395 (64.5)	765 (75.6)	
BMI, N (%)			0.0273			0.5972
< 25 kg/m^2^	296 (63.0)	756 (57.1)		369 (59.9)	622 (61.2)	
≥ 25 kg/m^2^	174 (37.0)	567 (42.9)		247 (40.1)	394 (38.8)	
Exercise, N (%)			0.7953			0.6643
< 150 min/week	431 (92.5)	1221 (92.9)		586 (96.2)	976 (96.6)	
≥ 150 min/week	35 (7.5)	94 (7.1)		23 (3.8)	34 (3.4)	
Sleep duration, N (%)			0.1283			0.0015
<7 hr/day	317 (67.4)	840 (63.5)		496 (80.5)	747 (73.6)	
≥7 hr/day	153 (32.6)	482 (36.5)		120 (19.5)	268 (26.4)	
Drinking status, N (%)			0.0276			0.0917
Never, past	108 (23.0)	372 (28.2)		430 (70.3)	750 (74.1)	
Current	362 (77.0)	946 (71.8)		182 (29.7)	262 (25.9)	
Volume of drinking, N (%)			0.9020			0.9730
< 200 g/week	360 (76.6)	1024 (77.4)		607 (98.7)	1000 (98.6)	
200–400 g/week	77 (16.4)	205 (15.5)		6 (1.0)	11 (1.1)	
≥ 400 g/week	33 (7.0)	94 (7.1)		2 (0.3)	3 (0.3)	
Smoking status, N (%)			0.0002			0.1114
Never smoker	93 (19.8)	332 (25.1)		594 (96.4)	989 (97.4)	
Ex-smoker	188 (40.0)	594 (44.9)		5 (0.8)	12 (1.2)	
Current smoker	189 (40.2)	397 (30.0)		17 (2.8)	14 (1.4)	
Volume of smoking, N (%)			< .0001			0.1419
No smoking	93 (19.9)	332 (25.2)		594 (96.6)	989 (97.5)	
< 10 pack-years	51 (10.9)	203 (15.4)		10 (1.6)	18 (1.8)	
10–20 pack years	79 (16.9)	266 (20.2)		5 (0.8)	5 (0.5)	
≥ 20 pack years	245 (52.3)	519 (39.3)		6 (1.0)	2 (0.2)	
Past history, N (%)						
Hypertension	22 (4.7)	70 (5.3)	0.6128	33 (5.4)	46 (4.5)	0.4517
Diabetes Mellitus	10 (2.1)	13 (1.0)	0.0574	8 (1.3)	13 (1.3)	0.9752
Hyperlipidemia	8 (1.7)	13 (1.0)	0.2112	17 (2.8)	17 (1.8)	0.1371
Carbohydrate intake (g/day)			0.4622			0.1243
Low	124 (26.6)	319 (24.3)		170 (27.7)	236 (23.3)	
Middle	234 (50.2)	658 (50.1)		293 (47.7)	523 (51.6)	
High	108 (23.2)	336 (25.6)		151 (24.6)	255 (25.2)	
Protein intake(g/day)			0.1864			0.0095
Low	128 (27.5)	316 (24.1)		179 (29.2)	228 (22.5)	
Middle	234 (50.2)	657 (50.0)		285 (46.4)	526 (51.9)	
High	104 (22.3)	340 (25.9)		150 (24.4)	260 (25.6)	
Fat intake(g/day)			0.8837			0.0040
Low	122 (26.2)	329 (25.1)		179 (29.2)	225 (22.2)	
Middle	230 (49.4)	654 (49.8)		282 (45.9)	535 (52.8)	
High	114 (24.5)	330 (25.1)		153 (24.9)	254 (25.0)	
Salt intake(g/day)			0.6514			0.4600
Low	124 (26.6)	321 (24.5)		163 (26.5)	244 (24.1)	
Middle	228 (48.9)	662 (50.4)		305 (49.7)	509 (50.2)	
High	114 (24.5)	330 (25.1)		146 (23.8)	261 (25.7)	
Potassium intake(g/day)			0.1562			0.0023
Low	129 (27.7)	315 (24.0)		179 (29.2)	228 (22.5)	
Middle	233 (50.0)	657 (50.0)		304 (49.5)	510 (50.3)	
High	104 (22.3)	341 (26.0)		131 (21.3)	276 (27.2)	

BMI: body mass index.

After adjusting for age, education, monthly income, BMI, sleep duration, volume of drinking, and smoking status, logistic regression analysis demonstrated that male heavy drinkers with depressive symptoms were 2.68 times more likely to have MetS than those without depressive symptoms (OR 2.68; 95% CI 1.11–6.49). Conversely, the odds ratio for prevalence of MetS in the high BMI group, compared to the lower BMI group, was 3.74 (95% CI 2.43–5.75; *p* for trend <0.001) in women with depressive symptoms. In men with depressive symptoms, the corresponding odds ratio (95% CI) for BMI was 6.02 (3.71–9.77) (Table 3).

**Table 3:** Adjusted odds ratios for metabolic syndrome in subjects with and without depressive symptoms.

	Men		Women	
Variable	With depressive symptoms	Without depressive symptoms	With depressive symptoms	Without depressive symptoms
Age	1.05 (1.01–1.09)	1.04 (1.01–1.06)	1.10 (1.06–1.13)	1.10 (1.07–1.13)
Education				
Middle school or less	1.00 (reference)	1.00 (reference)	1.00 (reference)	1.00 (reference)
High school or more	1.39 (0.62–3.11)	1.20 (0.71–2.04)	0.51 (0.31–0.86)	1.06 (0.68–1.68)
Monthly income				
≥ 1,500,000	1.00 (reference)	1.00 (reference)	1.00 (reference)	1.00 (reference)
< 1,500,000	1.12 (0.61–2.09)	1.54 (1.02–2.34)	0.94 (0.56–1.56)	0.82 (0.53–1.26)
BMI				
< 25 kg/m^2^	1.00 (reference)	1.00 (reference)	1.00 (reference)	1.00 (reference)
≥ 25 kg/m^2^	6.02 (3.71–9.77)	5.09 (3.83–6.77)	3.74 (2.43–5.75)	5.14 (3.60–7.34)
Exercise				
≥ 150 min/week	1.00 (reference)	1.00 (reference)		
< 150 min/week	1.65 (0.65–4.21)	1.48 (0.83–2.65)		
Sleep duration				
≥ 7 hour/day	1.00 (reference)	1.00 (reference)	1.00 (reference)	1.00 (reference)
< 7 hour/day	0.95 (0.58–1.54)	0.76 (0.58–1.01)	0.88 (0.50–1.52)	0.92 (0.62–1.37)
Drinking status				
Never, past			1.00 (reference)	1.00 (reference)
Current			1.57 (0.96–2.56)	0.95 (0.62–1.44)
Volume of drinking				
< 200 g/week	1.00 (reference)	1.00 (reference)		
200–400 g/week	0.86 (0.45–1.63)	1.18 (0.81–1.73)		
≥ 400 g/week	2.68 (1.11–6.49)	1.64 (1.00–2.69)		
Smoking status				
Never smoker	1.00 (reference)	1.00 (reference)	1.00 (reference)	1.00 (reference)
Ex-smoker	1.02 (0.53–1.94)	1.31 (0.93–1.86)	0.94 (0.09–9.84)	1.92 (0.40–9.11)
Current smoker	1.60 (0.84–3.05)	1.22 (0.83–1.81)	0.78 (0.19–3.23)	1.00 (0.18–5.50)
Carbohydrate intake (g/day)				
Low	1.00 (reference)	1.00 (reference)	1.00 (reference)	1.00 (reference)
Middle	1.03 (0.56–1.89)	0.85 (0.58–1.24)	1.65 (0.93–2.96)	1.11 (0.68–1.81)
High	0.79 (0.35–1.81)	0.71 (0.44–1.14)	1.80 (0.83–3.89	1.84 (1.00–3.41)
Protein intake(g/day)				
Low	1.00 (reference)	1.00 (reference)	1.00 (reference)	1.00 (reference)
Middle	1.37 (0.64–2.96)	1.34 (0.84–2.13)	0.84 (0.40–1.76)	0.68 (0.37–1.27)
High	1.30 (0.42–4.03)	1.69 (0.86–3.33)	1.43 (0.47–4.36)	1.23 (0.49–3.04)
Fat intake(g/day)				
Low	1.00 (reference)	1.00 (reference)	1.00 (reference)	1.00 (reference)
Middle	0.94 (0.46–1.92)	1.14 (0.76–1.71)	1.13 (0.60–2.12)	0.74 (0.44–1.24)
High	0.83 (0.32–2.15)	0.93 (0.53–1.62)	0.50 (0.20–1.22)	0.43 (0.20–0.93)
Salt intake(g/day)				
Low	1.00 (reference)	1.00 (reference)	1.00 (reference)	1.00 (reference)
Middle	0.97 (0.53–1.78)	0.89 (0.62–1.29)	1.68 (0.93–3.01)	1.12 (0.67–1.87)
High	1.44 (0.66–3.11)	1.02 (0.64–1.62)	1.22 (0.57–2.90)	1.55 (0.84–2.86)
Potassium intake(g/day)				
Low	1.00 (reference)	1.00 (reference)	1.00 (reference)	1.00 (reference)
Middle	0.82 (0.41–1.64)	0.79 (0.52–1.20)	1.13 (0.59–2.17)	1.79 (0.99–3.21)
High	0.94 (0.36–2.45)	0.88 (0.50–1.54)	1.18 (0.48–2.90)	1.53 (0.72–3.25)

## Discussion

We examined the association of lifestyle factors with the risk of MetS in individuals with depressive symptoms. After adjusting for variables, logistic regression analysis demonstrated that male heavy drinkers with depressive symptoms were 2.75 times more likely to have MetS than those without depressive symptoms (OR 2.75; 95% CI 1.16–6.53), while depressed women with a high BMI were 3.70 times more likely to have hypertension than those with lower BMI (OR 3.70; 95% CI 2.41–5.67).

Metabolic syndrome can be exacerbated by fat accumulation around intra-abdominal sites, organ impairment (e.g., nonalcoholic fatty liver disease), and exposure to xenobiotics [12]. The causes for elevation of individual risk factors are multiple and complex, and we do not yet have a clear understanding of how these risk factors combine to worsen the disease. Mitochondrial dysfunction seems to play a role in MetS and worsens as the disease progresses from insulin resistance to type 2 diabetes, and from nonalcoholic fatty liver disease to nonalcoholic steatohepatitis [13]. It is known that some drugs and environmental contaminants can lead to MetS or associated diseases. A study showed that metformin reduced the risk of MetS in patients with elevated fasting plasma glucose concentrations, although lifestyle changes were more effective [14]. Metformin has been shown to alter the intestinal microbiota, again suggesting the bacteria and viruses that populate the gut may play an essential role in MetS and associated diseases [15]. Environmental contamination may also be inherent in disease development, and meta-analysis of experimental evidence showed that dichlorodiphenyltrichloroethane (DDT) and dichlorodiphenyldichloroethylene (DDE) increased adiposity, while impairing thermogenesis and lipid dynamics in several mammalian species exposed to a range of doses [16]. These findings showed that prenatal exposure to DDT decreases heat production, and alters carbohydrate and lipid metabolism, thus making adult female offspring more likely to develop MetS [12]. Therefore, a multidisciplinary approach to understanding the underlying biological mechanisms and translation of this knowledge into prevention and treatment strategies are required [12].

Lifestyle is a well-known environmental factor that plays an essential role in promoting the development of MetS, or eventually aggravating its consequences [17]. Several previous studies have shown that a healthy lifestyle, defined by a combination of modifiable factors, was associated with up to roughly an 80% reduction in coronary heart disease (CHD) incidence [18–20] and a 50% decrease in ischemic stroke incidence [21] in white populations from developed countries. However, little is known about whether such protective effects are present in other non-white populations. Furthermore, epidemiological studies have shown that habitual consumption of dairy products may reduce the risk of MetS and, in particular, that dairy fat may have protective properties for attenuating the development of MetS in adult and elderly populations [22]. In this study, habitual consumption of dairy products, such as dairy fat intake and protein intake, was not associated with decreased in MetS. Park [23] suggested that alcohol had no effect on MetS in nonsmokers and light/moderate smokers. In heavy smokers, the risk of MetS was substantially higher in ex-drinkers, responsible drinkers, and hazardous drinkers compared to lifetime abstainers. However, we showed that male heavy drinkers who had depressive symptoms were associated with a higher risk of MetS, regardless of smoking status. In Korean adults, biased BMI and body image perception are associated with increased incidence of depression. Furthermore, specific weight control methods, such as fasting, skipping meals, and reducing food consumption rather than exercise, are associated with increased incidence of depression [24]. However, we showed that BMI was associated with increased risk of MetS in both men and women, regardless of the depressive symptoms.

In conclusion, this study suggests that heavy alcohol consumption might be associated with an increased risk of MetS in populations with depressive symptoms and BMI might be associated with a high risk of MetS, regardless of depression. Further well-designed, large-scale studies in other populations are warranted to validate our results. Therefore, a multidisciplinary approach to understand the underlying biological mechanisms and a better understanding of prevention and treatment are required.

### Limitations

The present study has some limitations that warrant consideration. First, participants were not analyzed over a follow-up period, and therefore, we could not individually evaluate whether the association between lifestyle factors and MetS was relevant in the long term. The baseline survey included 10,030 adults aged 40–69 years, and a total of seven traces were investigated. The Beck Depression Inventory (BDI) was only investigated in the second follow-up survey. We included 7,515 individuals who participated in the second follow-up survey. Among them, we excluded 4,079 participants with missing BDI data and more than half the participants have not BDI. Second, our findings do not apply to other populations, especially younger age groups of different ethnicities. Also, the cross-sectional nature of study has limited power to make conclusions about causality. However, this study showed a significant association between lifestyle factors such as BMI and MetS risk in adults with depressive symptoms. Lifestyle factors might be a useful marker to predict the disease modality and progression of chronic metabolic diseases, such as MetS, in clinical practice.
